# Safety Surveillance of Covishield Vaccine-Associated Adverse Events During the COVID-19 Pandemic: A Retrospective Longitudinal Study

**DOI:** 10.7759/cureus.67257

**Published:** 2024-08-19

**Authors:** Shilpa Chaudhary, Monica Aggarwal, Puja Kumari, Gopal Vishwas, Girish G Meshram, Rohit Dhaka, Minakshi Garg

**Affiliations:** 1 Pharmaceutical Sciences, Delhi Pharmaceutical Sciences and Research University, New Delhi, IND; 2 Pharmacology/Pharmacovigilance, Employees' State Insurance Corporation (ESIC) Medical College and Hospital, Faridabad, IND; 3 Pharmacology, Employees' State Insurance Corporation (ESIC) Medical College and Hospital, Faridabad, IND; 4 Clinical Medicine, Indian Council of Medical Research - Rajendra Memorial Research Institute of Medical Sciences (ICMR-RMRIMS), Patna, IND; 5 Pharmacology, Maulana Azad Medical College, New Delhi, IND; 6 Community Medicine, Employees' State Insurance Corporation (ESIC) Medical College and Hospital, Faridabad, IND; 7 Drug Regulatory Affairs, Delhi Pharmaceutical Sciences and Research University, New Delhi, IND

**Keywords:** patient safety, pharmacovigilance, aefi, covishield, covid-19

## Abstract

Background: Adverse events following immunization (AEFI) must be reported and assessed to promote patient safety. This longitudinal study examined the nature and severity of adverse events reported after Covishield (Serum Institute of India, Pune, India) vaccine administration to North Indians in a tertiary care hospital.

Method: A retrospective evaluation of adverse drug reactions (ADRs) reported after Covishield vaccine administration in our hospital over 18 months was conducted. The assessment was carried out to analyze the pattern of ADRs reported by individuals receiving the Covishield vaccine from January 2021 to June 2022. Data such as age, gender, category, dose administered, type of ADR, duration of the event, medical history, and outcome of the reactions were collected. Each reported adverse event was assessed individually. Causality was determined using the WHO-UMC causality assessment scale. The data were analyzed and are expressed as mean ± standard deviation and percentage.

Results: A total of 14,590 individuals were vaccinated at our study center from January 2021 to June 2022. During this period, 146 AEFIs (1.0%) were reported at our ADR monitoring center, Employees' State Insurance Corporation Medical College and Hospital (ESIC MCH), Faridabad, India. The majority of AEFIs were systemic, were reported after the first dose, and had an onset within 12 hours after vaccination. Fever, injection site pain, drowsiness, headache, vomiting, swelling, tenderness, and body aches were the most commonly reported adverse effects. No significant relationships were observed between the administered vaccine dose and sex, severity, duration of the event, or outcome. However, the incidence of adverse events was greater with the first vaccine dose than with the second dose. The possibility of serious or fatal adverse events was lowest in the general population and higher in the elderly with comorbidities.

Conclusion: The data suggest that the Covishield vaccine had mild to moderate adverse effects on the study population. This pharmacovigilance study will complement safety data and aid in the benefit-risk analysis of adverse effects associated with the Covishield vaccine. Additionally, healthcare professionals should be encouraged to conduct further safety studies by establishing robust vaccine safety monitoring systems in hospitals. Continuing medical education and workshops should also be conducted to educate healthcare workers about active surveillance.

## Introduction

The World Health Organization (WHO) declared coronavirus disease 2019 (COVID-19) a global pandemic due to its worldwide impact, affecting more than 220 countries [1,2]. COVID-19 is an infectious disease caused by severe acute respiratory syndrome coronavirus 2 (SARS-CoV-2) that first emerged in late 2019 when a cluster of severe pneumonia cases of unknown cause was reported in Wuhan, Hubei Province, China, and soon became a global threat [3,4]. Based on WHO dashboards, there are 500 million confirmed cases globally as of April 2022, and death counts exceed 6.2 million worldwide [5]. All regulatory authorities worked collaboratively by adopting various responsive measures, such as lockdowns, social distancing, the use of masks, and subsequent sanitization procedures, to prevent the spread of COVID-19 infection [6]. Pharmacological agents such as prophylactic anticoagulants, hydroxychloroquine, oxygen supplements, parenteral steroids, and other antiviral drugs, such as remdesivir, lopinavir/ritonavir, favipiravir, and oseltamivir, were used in the management of this pandemic, but none of these agents were 100% effective in seriously ill patients with high mortality [7].

This finding emphasized the need for the development and authorization of specific antivirals against SARS-CoV-2 to control the pandemic. Many pharmaceutical companies have developed vaccines against COVID-19 using preexisting and novel strategies, such as virus-vector vaccines, inactivated virus vaccines, messenger RNA (mRNA) vaccines, live attenuated virus vaccines, and protein subunit vaccines, to control the transmission of SARS-CoV-2 infection and reduce mortality [8]. Regulatory authorities in many countries approved the AstraZeneca vaccine from Oxford University, the mRNA vaccine from Moderna, and the mRNA-based BNT162b2 from Pfizer for emergency use [9]. In India, two vaccines were authorized by the Indian Drug Regulatory-Central Drug Standard Control Organization (CDSCO) for emergency use, namely Covishield from the Serum Institute of India (SII) and Covaxin from Bharat Biotech. Covishield (ChAdOx1-nCOV) is a genetically engineered, recombinant, replication-deficient chimpanzee adenovirus vector strain, whereas Covaxin consists of an inactivated SARS-CoV-2 viral particle. On January 16, 2021, India started vaccinating against SARS-CoV-2, first involving healthcare workers (HCWs) and other frontline workers, then extending to elderly people with different comorbidities, and finally to all age groups [10]. The Covishield vaccine, which is similar to the Oxford AstraZeneca vaccine concerning safety and immunogenicity, has accounted for almost 88% of all doses administered in the country and is the only vaccine available in our area [11]. Data from four clinical trials demonstrated that the Covishield vaccine has an efficacy of 67% (95% confidence interval) in preventing symptomatic COVID-19 infection and approximately 72%-100% in preventing hospitalization and severe infection beginning 21 days after receiving the second dose [12].

Current evidence on the safety profiles of COVID-19 vaccines relies mainly on data available from controlled trials and vaccine safety surveillance systems. According to a phase 2/3 clinical study conducted in India, the frequency of adverse events due to the Covishield vaccine was greater than 1%, including injection-site pain, pyrexia, body ache, headache, myalgia, malaise, asthenia, and fatigue [13]. The safety surveillance data for certain age groups, such as children, pregnant women, and individuals with comorbid conditions, have not been well studied. Although adverse events reported in clinical trials after post-marketing approval are common or uncommon ADRs, delayed-onset drug reactions will require extended pharmacovigilance studies [14]. Many more clinical studies will be required to justify the benefit-risk analysis of the vaccine over time. Therefore, this study is concerned with the safety surveillance of the Covishield vaccine (either first or second dose) by determining the number of adverse events and their association with related factors such as age, gender, category, severity (serious or non-serious), duration of the event, causality, and outcome in the North Indian population.

## Materials and methods

Study design: a noninterventional, retrospective observational study

Study Site and Study Population

This study was conducted in the Department of Pharmacology, Employees' State Insurance Corporation Medical College and Hospital (ESIC-MCH), a tertiary care hospital located in Delhi-NCR (Faridabad), India. Prior approval from the Institutional Ethics Committee was obtained (reference number: 134X/11/13/2022-IEC/05). Individuals who were vaccinated with the Covishield vaccine from January 2021 to June 2022 at ESIC Medical College & Hospital, Faridabad, and experienced adverse events were included in the study. The study site is a designated Regional Training Centre for Pharmacovigilance cum Adverse Drug Reaction Monitoring Centre (RTC-AMC) under the Pharmacovigilance Programme of India. All reported drug reactions were shared with the National Coordination Centre-Pharmacovigilance Programme of India (NCC-PvPI), Indian Pharmacopeia Commission, and Ghaziabad using Vigiflow software. In addition, serious adverse events were reported to the State Extended Program-On-Immunization Office (SEPIO) and the District Immunization Office (DIO) for further evaluation.

Informed Consent Statement

This retrospective study was performed by collecting data during routine ADR monitoring at the study site, so patient consent was waived. All study records were kept confidential at our ADR monitoring center, and the identities of the participants were not revealed in any unauthorized way.

Adverse Event Following Immunization (AEFI)

At the vaccination site, records were maintained, including demographic details (name, address, date of birth/age, gender, Aadhar number (a unique identification number issued by the Indian government), and mobile number) of all individuals who received either the first or second vaccine dose. Training was provided to the immunization staff through sensitization sessions to report any adverse events related to the Covishield vaccine and to notify the pharmacovigilance personnel to complete the Suspected Adverse Drug Reaction Reporting form (SUSAR; version 1.3). The contact details of the AMC (Adverse Drug Reaction Monitoring Centre) staff were also displayed at the vaccination center to facilitate the reporting process. The pharmacovigilance professionals also visited the vaccination site and emergency wards during the vaccination drive to collect ADRs. Additionally, from the records of vaccine recipients, they were followed up by pharmacovigilance personnel through phone calls for the occurrence of any AEFI. However, the majority of cases reported were spontaneous, while some cases were reported by follow-up.

Seriousness/Non-seriousness Criteria

The reported adverse events were considered serious if they resulted in any of the following outcomes: death, required hospitalization or prolonged hospitalization, or caused any birth defect or disability. Other than serious adverse events (SAEs), any reaction occurring was considered under non-serious criteria.

Data Collection and Causality Assessment

All adverse drug reactions reported in the ADR Monitoring Centre over 18 months were compiled and analyzed in this study. The relationship between adverse events and vaccine dose was established by Causality Assessment Committee (CAC) members with the help of the WHO-UMC Causality Assessment Scale [15].

Statistical Analysis

The data were analyzed for demographic variables (age, gender, dose, category, comorbidity status, previous history of COVID-19), type of adverse event (either serious/non-serious), duration of event, and outcome, which included recovered/recovering or fatal cases. Descriptive analysis was performed using IBM SPSS Statistics for Windows, Version 25 (Released 2017; IBM Corp., Armonk, New York). Age is expressed in mean values, and the number of adverse events, gender, category, dose administered, duration of event, medical history, and outcome of the reactions are expressed as percentages. The chi-square test and p-value were used to determine the association of adverse events with gender, category, severity, duration of AEFI, causality, and outcome. Variables with p < 0.05 were considered statistically significant.

## Results

Covishield vaccine administration started on January 22, 2021, at our study site. All vaccine recipients were older than 18 years of age. A total of 14,590 Covishield vaccine doses were administered during the study period from January 18, 2021, to June 30, 2022. Of these, 8,296 recipients received the first vaccine dose, and 6,294 received the second dose. During the study period, a total of 146 recipients reported adverse events, accounting for an AEFI of only 1.00%. The baseline characteristics and comorbidity statuses of all recipients reporting AEFIs are shown in Table 1. The non-serious AEFIs reported after the first and second doses of the vaccine were 97 (0.66%) and 38 (0.26%), respectively, with a recovery rate of less than three days. Out of seven (0.04%) serious adverse events, two (0.01%) AEFIs (one reported with symptoms of severe vomiting and diarrhea within six hours and the other reported with abdominal swelling followed by multiple skin lesions within 12-24 hours of the first vaccine dose) caused prolonged hospitalization. Both events were related to cerebrovascular accidents (CVAs), which led to the death of the recipients after four to five days of hospitalization and were unlikely related to the administered vaccine dose. The causal power of all AEFIs after the first and second vaccine doses was assessed with the WHO-UMC scale as probable (96 (0.65%); 38 (0.26%)), possible (7 (0.04%); 3 (0.02%)), or unlikely (2 (0.01%)).

**Table 1:** Baseline characteristics and comorbidity status of recipients reporting adverse events following immunization. *Total number of recipients reporting adverse events. **Total number of vaccine recipients.

Characteristic	Category	Frequency, n* (%)	% of Total Doses (N**=14,590)
Gender	Male	79 (54.11)	0.54
	Female	67 (45.89)	0.46
Dose distribution	First dose	104 (71.23)	0.71
	Second dose	42 (28.76)	0.29
Category	HCWs	70 (52.05)	0.48
	Non-HCWs	76 (47.95)	0.52
History of medical disorder	Yes	12 (3.42)	0.03
	No	141 (96.58)	0.97
History of COVID-19 infection	Yes	104 (71.23)	0.71
	No	42 (28.77)	0.29
Total	-	146 (100)	1.0

**Figure 1:**
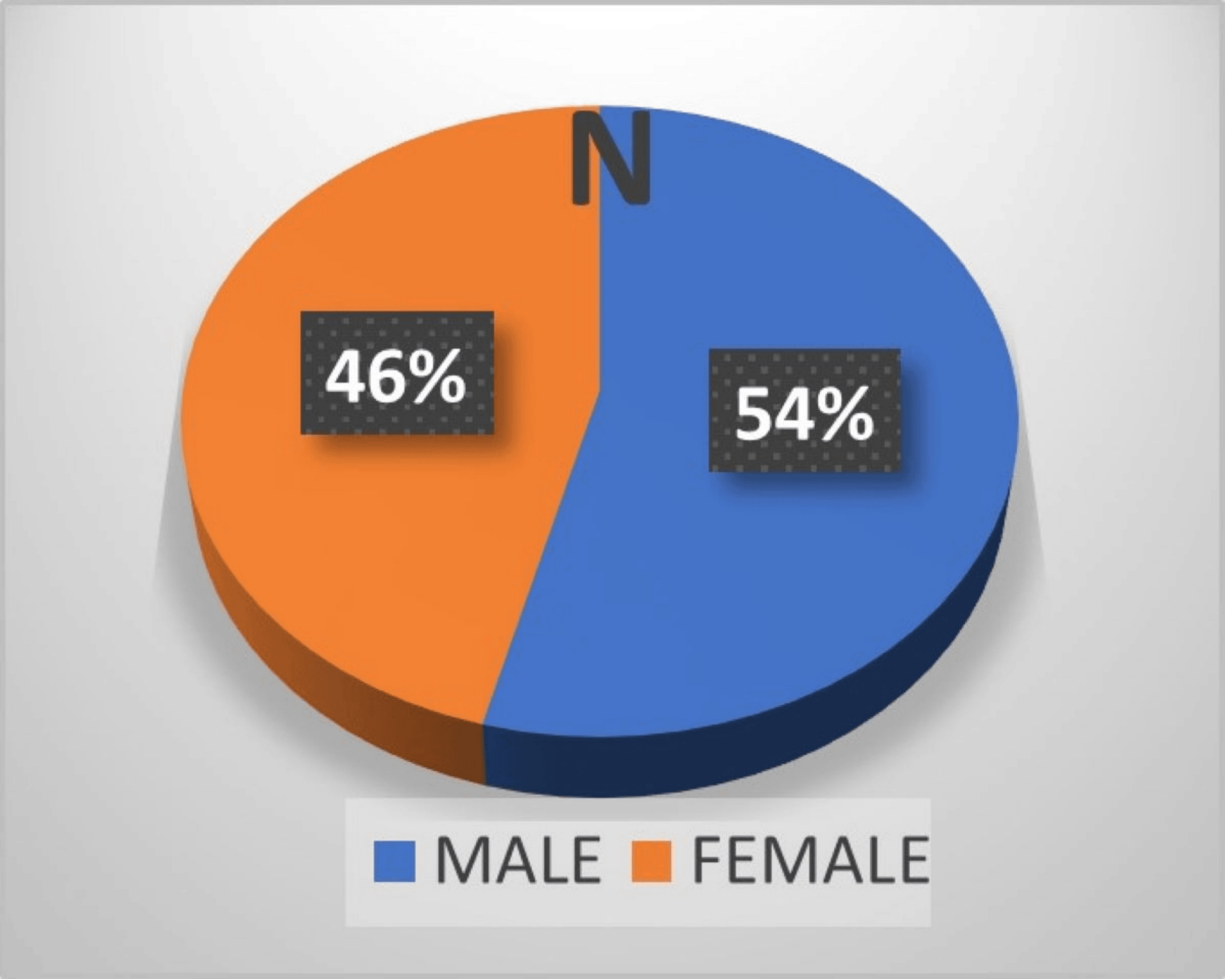
Distribution of males and females according to reported adverse events following immunization. Values are rounded.

Most of the AEFIs, 104 (71.23%), were reported after the first dose of the vaccine, while only 42 (28.76%) were reported after the second dose. Among the 42 recipients who had AEFIs after the second dose, 36 recipients had a history of AEFI after the first dose, but no one reported AEFI after the first dose due to a lack of awareness about reporting ADRs. A total of 79 (54.11%) of the AEFI patients were male, while 67 (45.89%) were female (Figure 1).

**Figure 2:**
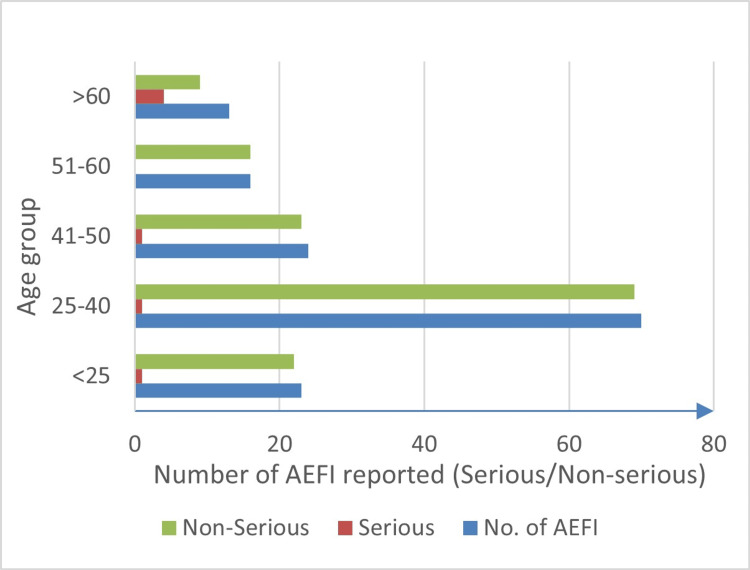
Mean age of recipients reported either serious or non-serious adverse events following immunization (AEFI).

The mean age of the recipients was 38.36 years (minimum 18 years and maximum 71 years). People in the age group of 25-40 years reported mild to moderate adverse drug reactions, whereas those older than 60 years reported the least but higher incidence of serious adverse events (Figure 2).

A total of 226 ADRs were observed among 146 recipients during the vaccination drive at the hospital. The majority of the ADRs involved generalized symptoms [108 (0.74%)], followed by pain [71 (0.48%)]. ADRs associated with other symptoms, such as gastrointestinal [13 (0.09%)], dermatological [11 (0.08%)], and respiratory [9 (0.06%)], were moderately reported. Adverse events related to the cardiovascular and central nervous systems [3 (0.02%)] were least reported among recipients (Table 2).

**Table 2:** System-wise distribution of Adverse events following immunization reported with Covishield vaccination. *n=226. One or more adverse drug reactions were shown in many recipients.

Characteristics	Category	Frequency of AEFI Reported in All Recipients (n*=226)		% Total Covishield Doses (N=14,590)
Generalized symptoms	Fever	79(54.1)	108	0.74
	Shivering and chills	12(8.2)		
	Weakness	12(8.2)		
	Malaise	5(3.4)		
Pain	Body ache	12(8.2)	71	0.48
	Myalgia	7(4.8)		
	Headache	22(15.1)		
	Limb pain/joint pain	7(4.8)		
	Injection site pain	23(15.8)		
Gastrointestinal symptom	Nausea	4(2.7)	13	0.09
	Vomiting	6(4.1)		
	Diarrhea	3(2.1)		
Central nervous system symptoms	Dizziness	3(2.1)	3	0.02
Dermatological symptoms	Itching	3(2.1)	11	0.08
	Rash	8(5.4)		
Respiratory symptoms	Cough	1(0.7)	9	0.06
	Cold	4(2.7)		
	Sore throat	2(1.4)		
	Breathlessness	2(0.7)		
Cardiovascular symptoms	Chest pain	2(1.4)	3	0.02
	Hypotension	1(0.7)		

**Figure 3:**
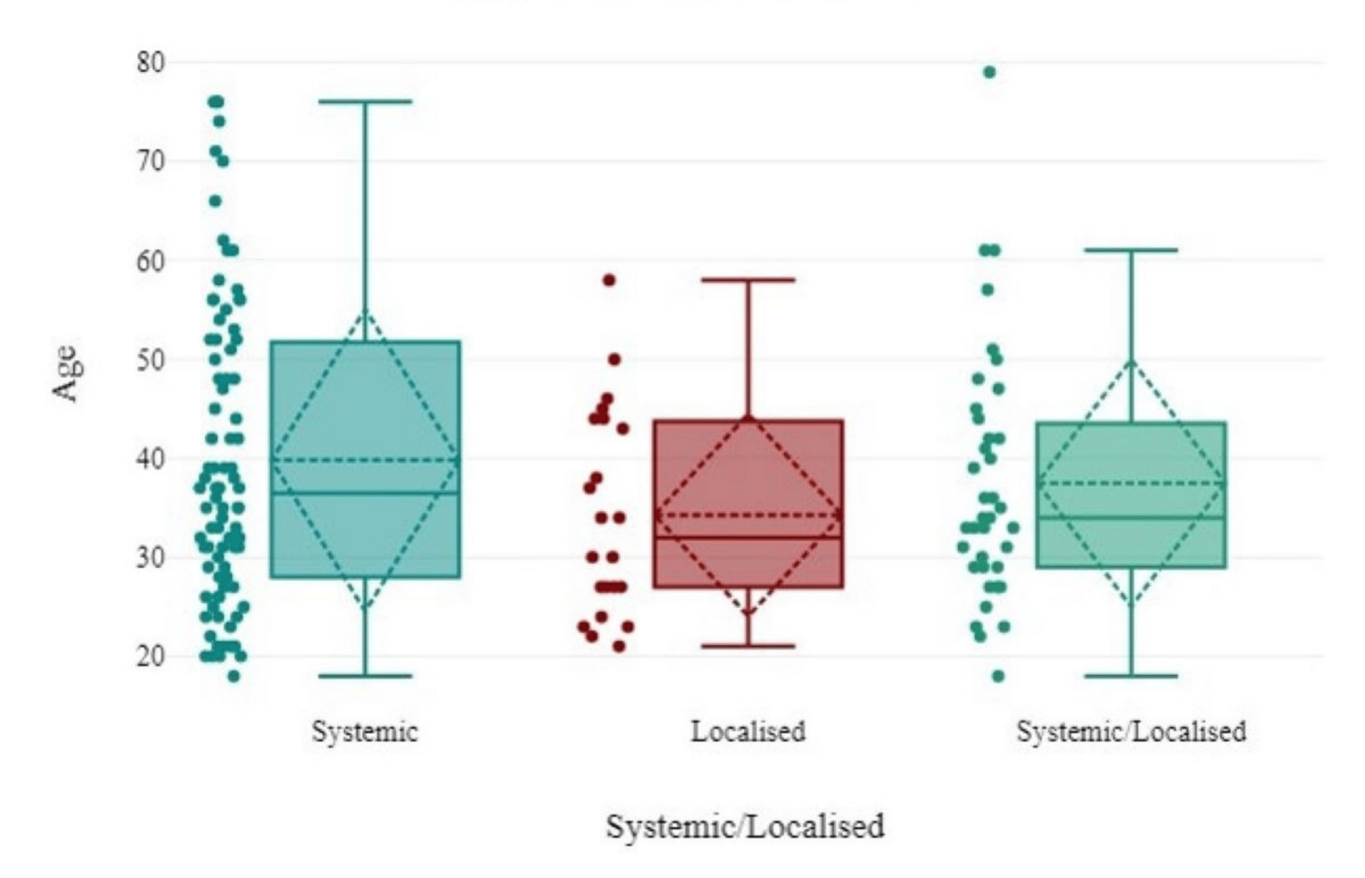
Relationship between systemic and localized drug events with age.

Only 12 recipients with comorbidities (diabetes, hypertension, hyperlipidemia, hyperthyroidism, asthma, paralysis of one arm, and allergy) experienced generalized, cardiovascular, gastrointestinal, dermatological, and central nervous system-related adverse events. In this safety surveillance data, we observed 86 (58.90%) systemic and 22 (15.06%) localized drug reactions, while 38 (26.02%) recipients presented with both systemic and localized symptoms, as shown in Figure 3.

Compared with other age groups, recipients between 28 and 52 years of age experienced the highest number of systemic drug reactions. Pain or tenderness at the injection site [23 (15.08%)] was the most common localized reaction reported by recipients after the first and second doses of vaccination. Among the systemic AEFIs, fever [144 (99.11%)], headache [22 (12.33%)], shivering with chills [12 (8.2%)], neuromuscular weakness [12 (8.2%)], body ache [12 (8.2%)], and myalgia [7 (4.8%)] were the most common adverse events. However, the frequency of adverse events was significantly greater after the first dose than after the second vaccine dose (Table 3).

**Table 3:** Comparison of the AEFI after the first and second vaccine doses. AEFI: adverse events following immunization.

AEFI	Dose 1	Dose 2	Total
Fever	65	14	79
Injection site pain	17	6	23
Shivering and chills	11	1	12
Neuromuscular weakness	7	5	12
Cough	1	0	1
Swelling	4	3	7
Itching	1	2	3
Vomiting and nausea	7	3	10
Dizziness	1	2	3
Diarrhea	3	0	3
Headache	16	6	22
Hypotension	1	0	1
Breathlessness	2	0	2
Running nose/cold	4	0	4
Joint pain	1	2	3
Sweating	1	0	1
Sore throat	2	0	2
Rash	5	3	8
Bodyache	7	5	12
Chest heaviness/chest pain	2	0	2
Malaise	3	2	5
Myalgia	5	2	7
Pain in limbs	2	2	4
Total	168	58	226

There was no significant relationship (p > 0.05) between the first or second dose of the vaccine and the development of AEFI, as indicated by the vaccine dose (χ²(1) = 1.44, p = 0.23; Cramer's V = 0.1); seriousness criteria (χ²(1) = 0.34, p = 0.563; Cramer's V = 0.05); duration of AEFI (χ²(2) = 1.91, p = 0.384; Cramer's V = 0.11); outcome (χ²(2) = 0.82, p = .664; Cramer's V = 0.07); or causality (χ²(2) = 0.13, p = 0.936; Cramer's V = 0.03). However, the number of adverse events reported with the first dose of vaccination was significantly greater than those reported with the second dose. Moreover, we found a statistically significant relationship between both vaccine doses and HCWs or non-HCWs (χ²(1) = 16.61, p < 0.001; Cramer's V = 0.34). After the first vaccine dose, HCWs reported more adverse events than non-HCWs, but after the second dose, non-HCWs reported more (Table 4).

**Table 4:** Comparison of risk factors for AEFI reported after the first and second doses. *p<0.05 was considered as statistically significant. AEFI: adverse events following immunization, HCW: healthcare worker.

Risk Factors		Frequency of AEFI Reported Due to Covishield, n (%N)		p-value*
		Dose 1	Dose 2	
Gender	Male	53(0.36)	26(0.17)	0.23
	Female	51(0.34)	16(0.10)	
HCWs/non-HCWs	HCWs	61(0.41)	09(0.06)	<0.001
	Non-HCWs	43(0.29)	33(0.22)	
Seriousness criteria	Serious	07(0.04)	04(0.02)	0.563
	Non-serious	97(0.66)	38(0.26)	
Duration of AEFI	<3 days	97(0.66)	38(0.26)	0.384
	>3 days	05(0.03)	04(0.02)	
Co-morbidity, if any	-	7(0.04)	5(0.03)	-
Outcome	Recovering	0	0	0.664
	Recovered	102(0.67)	42(0.28)	
	Fatal	02(0.01)	0	
Causality	Certain	0	0	0.936
	Probable	96(0.65)	38(0.26)	
	Possible	07(0.04)	03(0.02)	
	Unlikely	02(0.01)	0	

The non-serious AEFIs reported after the first and second doses of the vaccine were 97 (0.66%) and 38 (0.26%), respectively, with a recovery rate of less than three days. Out of seven (0.04%) serious adverse events, two (0.01%) AEFIs (one reported with symptoms of severe vomiting and diarrhea within six hours and the other reported with abdominal swelling followed by multiple skin lesions within 12-24 hours of the first vaccine dose) caused prolonged hospitalization. Both events were related to cerebrovascular accidents (CVAs), which led to the death of the recipients after four to five days of hospitalization and were unlikely related to the administered vaccine dose. The causal power of all AEFIs after the first and second vaccine doses was assessed with the WHO-UMC scale as probable (96 (0.65%); 38 (0.26%)), possible (7 (0.04%); 3 (0.02%)), or unlikely (2 (0.01%)).

## Discussion

As per reports of Indian national data, 1.51 billion doses of vaccination against COVID-19 were administered through January 2022, and causality was assessed only for serious cases [16]. According to nationwide data from India, a total of 49,819 adverse events were reported from 1.23 billion doses of the COVID-19 vaccine, of which 47,691 were nonserious cases; 163 were severe, and 1,965 cases were reported as serious, with 4 per 100,000 doses or 0.004% [17]. However, in other countries, due to the Covishield (AstraZeneca) vaccine, AEFI rates amounted to 107.21 per 100,000 doses in Canada, 76.5 per 100,000 doses in the countries of the Americas, 20.40 per 100,000 doses in Mexico, and 17.45 per 100,000 doses in Brazil [18-20]. The causality assessment of serious adverse events reported 946 vaccine-related deaths from all COVID-19 vaccines (Covishield, Covaxin, and Sputnik), whereas European countries reported 938 fatal adverse events from 58.4 million doses of the Covishield vaccine alone [16]. At our study center, although the total vaccination rate with Covishield was comparatively low, we observed a higher AEFI rate, i.e., 1.0% of the total vaccination. The higher AEFI rates could be due to the increased reporting of adverse events by healthcare professionals at vaccination sites and routine visits by RTC-AMC staff in OPDs/wards.

In the present retrospective analysis, we observed AEFIs among 146 vaccine recipients receiving 14,590 doses of Covishield (refer to Table 1). The most common systemic adverse events reported were fever, pain at the injection site, headache, body ache, and myalgia (refer to Table 2). Similar findings were also observed in Rahat et al.’s study [6], which noted that pain at the injection site followed by fever and malaise were the most common adverse events reported with the Covishield vaccine. The study conducted by Sathyapalan et al. [7] reported a higher incidence of symptoms such as fever, sore throat, headache, vomiting, and nausea in females compared to males. This study compares the reported AEFIs between the first and second vaccine doses according to gender, category (HCP and non-HCP), duration of AEFI (recovery rate), severity, outcome, and causality. We found no association between developing AEFI and gender with either vaccine dose. However, the number of adverse events reported in males and females after the first dose was greater than after the second dose (refer to Table 4). 

In contrast, the adverse events reported by Mittal et al. [21] indicated that although male recipients (57%) were more affected than female recipients (43%) with the Covishield vaccine, the reporting rate was higher after the second dose than after the first dose. However, a significant association (p < 0.001) was observed between events reported by HCPs and non-HCPs for either vaccine dose. Compared with non-HCWs, HCWs reported a greater incidence of AEFI after the first dose (refer to Table 4). This can be explained by the fact that at the beginning of the vaccination drive at the study site, only HCWs received the vaccine, and due to active encouragement of reporting ADRs, AEFIs were reported. Otherwise, there was no direct association of adverse events found in previous studies with gender or category. However, our study demonstrated a decreased risk of developing adverse events with age; i.e., patients aged 18-40 years had a higher occurrence of non-serious adverse events than those aged over 40 years (refer to Figure 2). Our findings regarding the development of fewer serious AEFIs with increasing age are similar to the findings of published clinical studies on the Covishield vaccine [22,23]. During the study period, only 12 recipients with comorbidities such as diabetes, hypertension, thyroid disease, hyperlipidemia, and asthma reported adverse events and presented delayed recovery of more than three days.

Limitations of the study

There are a few limitations in our study. This study was based on self-reported data, which could be influenced by participants’ preconceptions of adverse events following the COVID-19 vaccine. Some recipients' data were missed due to a lack of contact details at the vaccination site, therefore they could not be followed up for AEFI. In this study, we only included the total number of men or women who experienced AEFI, but we did not have information about the total number of men and women who received the vaccination. This study represented several strong points, such as long-term safety surveillance of the Covishield vaccine in a tertiary care hospital and findings of serious adverse events with fatal outcomes. We reported all possible adverse drug reactions, both serious and non-serious, based on causality assessment during vaccination. Moreover, this safety surveillance data helps to investigate the benefit-risk analysis of the Covishield vaccine in the future.

## Conclusions

Our study demonstrated that the Covishield vaccine was safe and well-tolerated with low reactogenicity in the general population, but it may produce mild to moderate or severe adverse events in the comorbid population. Non-serious adverse drug reactions may develop in all age groups, but people above 60 years of age are at a higher risk of developing serious adverse events. To further validate these findings, many more clinical studies should be conducted in tertiary care hospitals to gather adverse event data for all newly approved vaccines or drugs in human use. Moreover, continuing medical education and workshops should be held to create awareness among healthcare professionals and consumers about reporting adverse events for better patient safety in the future.
